# Nociceptive TRP Channels: Sensory Detectors and Transducers in Multiple Pain Pathologies

**DOI:** 10.3390/ph9040072

**Published:** 2016-11-14

**Authors:** Aaron D. Mickle, Andrew J. Shepherd, Durga P. Mohapatra

**Affiliations:** 1 Department of Anesthesiology, Washington University School of Medicine, 660 S. Euclid Avenue, St. Louis, MO 63110, USA; amickle@wustl.edu (A.D.M.); a.shepherd@wustl.edu (A.J.S.); 2 Washington University Pain Center, Department of Anesthesiology, Washington University School of Medicine, 660 S. Euclid Avenue, St. Louis, MO 63110, USA; 3 Center for Investigation of Membrane Excitability Diseases, Washington University School of Medicine, 660 S. Euclid Avenue, St. Louis, MO 63110, USA; 4 Siteman Cancer Center, Washington University School of Medicine, 660 S. Euclid Avenue, St. Louis, MO 63110, USA

**Keywords:** TRP channel, pain, nociception, inflammatory pain, neuropathic pain, visceral pain, cancer pain, migraine, dental pain, peripheral sensitization

## Abstract

Specialized receptors belonging to the transient receptor potential (TRP) family of ligand-gated ion channels constitute the critical detectors and transducers of pain-causing stimuli. Nociceptive TRP channels are predominantly expressed by distinct subsets of sensory neurons of the peripheral nervous system. Several of these TRP channels are also expressed in neurons of the central nervous system, and in non-neuronal cells that communicate with sensory nerves. Nociceptive TRPs are activated by specific physico-chemical stimuli to provide the excitatory trigger in neurons. In addition, decades of research has identified a large number of immune and neuromodulators as mediators of nociceptive TRP channel activation during injury, inflammatory and other pathological conditions. These findings have led to aggressive targeting of TRP channels for the development of new-generation analgesics. This review summarizes the complex activation and/or modulation of nociceptive TRP channels under pathophysiological conditions, and how these changes underlie acute and chronic pain conditions. Furthermore, development of small-molecule antagonists for several TRP channels as analgesics, and the positive and negative outcomes of these drugs in clinical trials are discussed. Understanding the diverse functional and modulatory properties of nociceptive TRP channels is critical to function-based drug targeting for the development of evidence-based and efficacious new generation analgesics.

## 1. Introduction

Pain constitutes an “unpleasant sensory and emotional experience associated with actual or potential tissue damage”, as defined by The International Association for the Study of Pain [1]. As a sensory modality, pain represents an integral part of life. It serves as a protective mechanism and an associative condition, as well as an alarm system for a wide range of pathological conditions. In addition, pathologies and/or disease conditions that exclusively lead to pain also exist. The first and foremost process is the peripheral detection and transduction of noxious stimuli that are determined as painful by the higher-order structures in the central nervous system (CNS). The terminology that has been widely used to define this process is “nociception”, which accounts for the neural mechanisms and pathways for the encoding and processing of noxious stimuli [1]. Nociception constitutes the primary physiological and/or pathophysiological process for the somatic, visceral and trigeminal sensory systems. The specialized receptive fields as well as the molecular entities therein are widely regarded as nociceptors. Sensory neurons of the peripheral nervous system (PNS) that transmit nociceptive signals lie in distinct populations of sensory ganglia. These neurons send peripheral afferents to the somatic, visceral, and craniofacial regions, and also connect to the spinal cord and brain stem. These neurons are the critical anatomical/structural mediators of sensory signal transmission between the PNS and CNS.

Nociceptive neurons are functionally characterized by the type of sensory receptors and ion channels expressed on the plasma membrane throughout the cell body (somata) and nerve fibers. These receptors/channels are vital for the detection of various noxious stimuli. These neurons also possess the molecular machinery to convert the noxious signals into electrical signals and transmit this information to the CNS. Nociceptive receptors/channels, membrane proteins belonging to the Transient Receptor Potential (TRP) family, constitute the major group of molecular detectors/ transducers. The first TRP channel discovered was a defective phototransduction channel from a mutant form of *Drosophila* that exhibited an abnormally transient membrane potential change in response to bright light, and was subsequently found preserved/conserved in many animal species [2,3]. TRPs are non-selective cation channels with relatively high Ca^2+^-permeability, and are expressed in a wide variety of cell/tissue types, both on the plasma membrane and intracellular organelle membranes [4,5]. They also share identical overall membrane topology, consisting of tetramers of 6-transmembrane (6-TM) segment polypeptide subunits with a central ion conduction pore, which is similar to voltage-gated K^+^ channels. Since their discovery, the TRP family of proteins have now grown significantly, and to date consist of six sub-families with 28 mammalian members; categorized as canonical (TRPC), vanilloid (TRPV), ankyrin (TRPA), melastatin (TRPM), polycystin (TRPP), and mucolipin (TRPML) [4,5]. In general, TRP channels are primary transducers of most known sensory modalities such as vision, hearing, olfaction, taste and touch, to a wide range of innocuous-to-noxious stimuli, and are therefore one of the most extensively studied receptor families in sensory biology [5,6,7]. The diversity in TRP channels is mainly linked to the greatest level of amino acid sequence differences in their cytoplasmic N- and C-termini. Based on their ability to detect and transduce specific nociceptive modalities, members of only three TRP sub-families, TRPV, TRPA and TRPM, have been grouped into the category of “nociceptive TRP channels”. Activation of nociceptive TRP channels by specific noxious and/or pain-producing stimuli serves as the principal mode of detection/transduction of pain under physiological and pathophysiological conditions. In addition, modifications in channel function and trafficking properties, as well as changes in gene expression of nociceptive TRP channels are considered to be highly critical for peripheral nociceptive and pain processing under a wide variety of pathological conditions. Since in-depth studies have already been conducted to characterize the role of nociceptive TRP channels in multiple pain and migraine pathologies, they constitute attractive targets for new-generation analgesics and anti-migraine drug developments [8,9,10,11,12,13]. This review summarizes a comprehensive knowledge on the molecular characterization of nociceptive TRP channels, their constitutive and modulatory functions, expression and tissue distribution, as well as how these channels and their specific properties are critically involved in various pain conditions. Recent developments in analgesic targeting of nociceptive TRP channels are also outlined here.

## 2. Nociceptive TRP Channels

### 2.1. History, Identification and Cloning

Excitation of sensory nerves by multiple chemical and physical stimuli was first described over 100 years ago [14]. In addition to heat and cold stimuli, one of the first chemical compounds described to activate sensory neurons was capsaicin. Extensive research studies conducted between 1960 and the mid-1990s showed specific actions of capsaicin on sensory neurons, and thereby proposed the existence of a specialized receptor—“the capsaicin receptor” [15]. Even before molecular cloning, studies suggested that the capsaicin receptor was a TRP-like receptor channel, since capsaicin’s actions on sensory nerves were effectively blocked by the non-selective TRP channel blocker ruthenium red [16]. Ultimately, in 1997 the molecular identity of the capsaicin receptor was revealed by expression cloning using a cDNA library generated from rodent sensory neurons [17], and named as “vanilloid receptor subtype-1” (VR1). Subsequently VR1 was assigned as the first member of the new TRP channel family TRPV, and referred to as TRPV1. Along with gene discovery, further characterization of TRPV1 revealed the ability of this channel to be activated by multimodal pain-producing stimuli, as well as integration of such stimuli at the channel protein level [17,18]. This discovery provided the much-awaited catalyst for the subsequent discovery of a series of nociceptive TRP channels for several noxious and painful stimuli. The next TRP channel cloned was TRPV2 and characterized as the high noxious temperature transducer on rodent sensory neurons [19]. Utilizing TRPV1 and TRPV2 cDNA sequences, the TRPV4 expressed-sequence tag (EST) from GenBank, and subsequently the cDNA for TRPV4 from rat kidney cDNA library were identified as osmotically-activated TRP channels (VR-OAC and OTRPC4) [20,21]. Similar approaches also led to the identification and cloning of TRPV5 and TRPV6 [22,23,24]. It was also proposed that cold temperatures and menthol, the cooling compound in mint leaves, activate another receptor on sensory neurons [25]. It was subsequently confirmed with the cloning and characterization of the “cold-menthol receptor-1”, later designated as TRPM8 [26,27]. With both heat- and cold-activation receptors identified, the obvious next question centered on the identity of receptor(s) that could be activated by temperatures in the neutral range. This led to the identification/cloning of TRPV3 from human sensory neurons and rodent keratinocytes, based on a sequence homology cloning strategy utilizing TRPV1–6 sequences [28,29]. Although originally cloned from human lung fibroblasts as a cell growth-controlling protein [30], identification and cloning of ankyrin-containing transmembrane protein-1 (ANKTM1), later designated as TRPA1, from rodent sensory neurons led to the discovery of a noxious cold- and mechano-sensitive channel [31]. Similarly, the EST of TRPM3 channel was initially cloned and characterized from a human kidney cDNA library, as a Ca^2+^ permeable channel, and subsequently as the receptor for several steroids, including pregnenolone sulfate [32,33,34]. Much later, the thermo-sensing and nociceptive properties of TRPM3 were identified and characterized [35]. Overall, the nociceptive TRP channel group represents a critical mass of receptors sensing diverse painful and non-painful modalities, and are thus extensively studied, as well as explored as therapeutic targets.

### 2.2. Characterization of Channel Function: Activation by Diverse Physico-Chemical Stimuli

**Figure 1:**
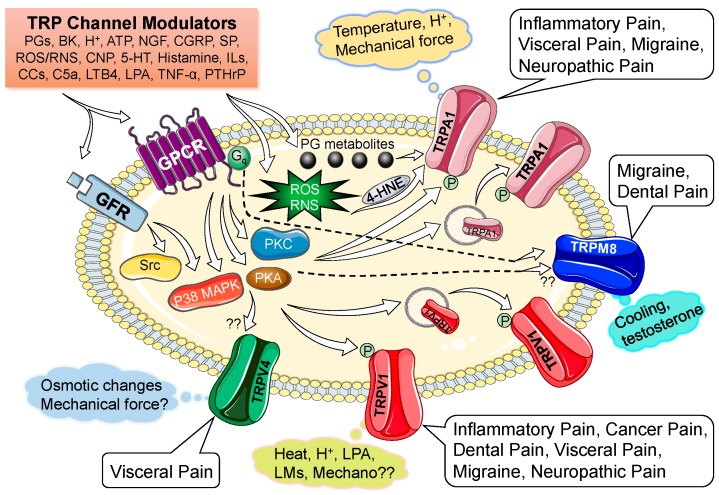
Scheme depicting specific activators and modulators of various nociceptive transient receptor potential (TRP) channels on mammalian sensory neurons. Involvement of individual TRP channels in specific pain-related pathologies are indicated/listed within call-out boxes. Abbreviations: 5-HT, serotonin; ATP, adenosine triphosphate; BK, bradykinin; CCs, chemokines; CGRP, calcitonin gene-related peptide; CNP, C-type natriuretic peptide; GFR, growth factor receptor; GPCR, G protein-coupled receptor; H^+^, protons; ILs, interleukins; LMs, lipid mediators; LPA, lysophosphatidic acid; LTB4; leukotriene B4; NGF, nerve growth factor; p38 MAPK, p38 mitogen-activated protein kinase; PGs, prostaglandins; PKA, cAMP-dependent protein kinase; PKC, protein kinase C; PTHrP, parathyroid hormone-related peptide; ROS, reactive oxygen species; RNS, reactive nitrogen species; SP, substance P or neurokinin; Src; *src*-type protein kinase; TRPA1, transient receptor potential sub-family ankyrin, member-1; TRPM8, transient receptor potential sub-family melastatin, member-8; TRPV1, transient receptor potential sub-family vanilloid, member-1; TRPV4, transient receptor potential sub-family vanilloid, member-4.

Distinct physico-chemical activation represents the crucial functional property of nociceptive TRP channels, a property that was exploited to the fullest to aid in their identification and cloning. In addition, algogenic chemicals found in natural sources, such as plants and spices, greatly influenced the functional characterization and provided identification of these channels. The majority of these channels turn out to be polymodal, in terms of their activation. The exhaustive list of exogenous and endogenous activators of nociceptive TRP channels and their underlying structure-functional mechanisms have recently been detailed [11]. Here we summarize only the endogenous or pathophysiological activators of nociceptive TRP channels (Figure 1). Starting with the major polymodal nociceptive TRP channel, TRPV1, which is directly activated by noxious temperatures (≥43 °C) and an acidic extracellular environment (~pH 6.0 or less) [9,17,18,36], as well as by a basic intracellular environment (~pH 7.8 or more) [37]. A number of endovanilloids and endocannabinoids generated by lipid metabolism pathways and/or under inflammatory conditions also directly activate TRPV1 [9,38,39,40]. In heterologous expression systems, TRPV1 can also be activated by reactive oxygen and nitrogen species (ROS/RNS), although whether such activation operates in sensory neurons still remains a matter of debate [41]. Activation of TRPV1 leads to the influx of Ca^2+^ and Na^+^ (with relatively high permeability for Ca^2+^) through the channel pore [17,42,43], which then results in neuronal plasma membrane depolarization and subsequent opening of Na_v_/Ca_v_ channels to initiate AP firing. Following activation, the TRPV1 channel undergoes rapid Ca^2+^-dependent desensitization, resulting in diminished AP firing [17,18,43,44,45]. In contrast, the TRPV2 channel can only be activated by higher noxious temperatures (>52 °C), with no endogenous ligands identified so far [19,34]. Activation of TRPV2 also leads to cellular influx of Ca^2+^ and Na^+^ with relatively high Ca^2+^ permeability [19]. Similarly, warm temperatures account for the activation of TRPV3 (32 °C to 40 °C) and TRPV4 (≥33 °C), which then lead to cellular influx of Ca^2+^ and Na^+^ with high relative Ca^2+^ permeability [5,46,47], resulting in membrane depolarization and subsequent AP firing. In addition, TRPV4 can be activated by osmotic changes and mechanical forces such as pressure and shear stress [47,48,49,50].

Although preliminary studies suggested pregnenolone sulfate was the endogenous activator of TRPM3, warm/noxious temperature (>30 °C) activation of the channel has also been shown subsequently [33,34,35]. Activation of TRPM3, like TRPVs, lead to cellular influx of Ca^2+^ and Na^+^ with relatively high Ca^2+^ permeability [33,35]. In contrast, TRPM8 can be activated by innocuous cooling (26 °C–15 °C) to noxious cold temperatures (15 °C–8 °C), leading to cellular influx of Ca^2+^ and Na^+^ with high relative Ca^2+^ permeability [26,27,51]. In addition, TRPM8 can be directly activated by testosterone in human prostate cell lines and rat DRG neurons [52,53].

TRPA1 was originally described as a cold-sensing ion channel [31,54,55], although others have debated this conclusion [56]. Comparative analysis of TRPA1 from different species showed that rodent, but not primate TRPA1 could be activated at noxious cold temperatures, which was directly linked to regions within the 5th transmembrane domain and pore region of the channel protein [57]. In contrast, a recent report suggests that human TRPA1 exhibits a U-shaped temperature-activation curve [58]. It shows robust channel activation at noxious cold temperatures (≤15 °C), relative inactivity at mild cooling temperatures (20 °C–25 °C), then increased channel opening at neutral-to-warm temperatures (25 °C–35 °C), and finally a decrease in channel open probability at noxious warm temperatures [58]. Earlier, mouse TRPA1 was shown to be insensitive to warm temperatures [59]. Similarly, acidic pH has also been shown to activate TRPA1 in a species-dependent manner; human, but not rodent TRPA1 is sensitive to acidic pH [60], and a dose-dependent increase in channel gating was found for increasing proton concentration associated with CO_2_ or other weak organic acids [61,62]. ROS, a redox mediator released during injury/inflammation activates TRPA1 via cysteine oxidation or formation of disulphide bonds between cysteine residues in the channel protein [63,64,65]. Furthermore, it has been suggested that ROS-mediated lipid peroxidation leads to 4-hydroxynonenal (4-HNE) production, which then covalently modifies the cytoplasmic cysteine residues to activate TRPA1 [64,66]. Reactive nitrogen species have also been shown to activate the channel by S-nitrosylation [63,64,65,67]. In addition to nitric oxide (NO), another “gasotransmitter”, hydrogen sulphide (H_2_S), has also been suggested to activate TRPA1 channel [68,69]. Also, prostaglandin (PG) metabolic products, such as 15dPGJ2, PGA2 and PGA1 have shown to directly activate TRPA1 [70,71]. Elevated levels of intracellular Ca^2+^ have been suggested to activate TRPA1 directly via interaction with one of the three Ca^2+^-binding EF-hand domains in the cytoplasmic N-terminus of the channel protein [72]. In addition to temperature and chemical activation, TRPA1 channel has also been shown to be a transducer of mechanical force [73,74], and mechanical activation of the channel has been suggested to be modulated by other algesic stimuli [75]. Like other nociceptive TRP channels, activation of TRPA1 also results in increased cellular influx of Ca^2+^ and Na^+^ with relatively high Ca^2+^ permeability, leading to sensory neuron membrane depolarization and subsequent AP firing. It has also been suggested that the Ca^2+^ permeability of TRPA1 increases with repeated agonist stimulation [76].

Taken together, a comprehensive scheme for pathological activation of nociceptive TRP channels in sensory neurons can be proposed. Tissue acidosis, which leads to increased H^+^ levels, can directly activate TRPV1 and TRPA1 (but only in humans). Similarly, inflammatory and tissue injury conditions produce elevated levels of H^+^, ATP, PG metabolites, ROS/RNS, HNO, H_2_S, and several lipid metabolites, which can lead to combined activation of TRPV1 and TRPA1 channels. Increased intracellular Ca^2+^ levels, due to activity of multiple TRP channels during pathological conditions, could further activate/potentiate TRPA1 channels. Furthermore, mechanical activation of one or more nociceptive TRP channels, TRPV4, TRPA1 and TRPV1, could be achieved due to the action of pro-algesic agents. Collectively, it is highly likely that multiple nociceptive TRP channels are activated during pathological conditions, which presumably constitute the mechanism for a stronger and long-lasting nociceptor excitation.

### 2.3. Similarities and Differences in Channel Expression and Localization

Most nociceptive TRP channels are predominantly expressed in small- and medium-diameter peripheral sensory neurons in the trigeminal ganglia (TG), dorsal root ganglia (DRG), sympathetic ganglia (SG) and nodose ganglia (NG). Significant expression of several nociceptive TRP channels has also been shown in the CNS and other tissue and cell types, such as keratinocytes, vascular endothelial cells, bladder epithelial cells and fibroblasts. Furthermore, TRPV1-3 have also been shown to be expressed in human dental pulp [9,11]. TRPV1 protein expression has been detected in peptidergic sensory afferents innervating bones [77], although expression of other nociceptive TRP channels there remains unexplored. Significant overlap between TRPV1 and TRPA1 expression in small/medium-diameter nociceptive neurons in the DRG and TG has been observed. In contrast, TRPM8 shows minimal overlap with TRPV1 and TRPA1 expression in these ganglia [9,78]. Expression of TRPV4 in DRG neurons has been a matter of debate, with initial reports showing functional TRPV4 expression in DRG neurons [50,79], and a subsequent report suggesting no functional TRPV4 expression in mammalian DRG neurons [80]. Information on expression of TRPV2-6, and TRPM3 channels in specific populations of sensory neurons and CNS neurons is still lacking. A recent surge in the utilization of transcriptome analysis has enabled unbiased, large-scale determination of gene expression signatures in DRG neurons from rodents and humans [81,82]. Furthermore, this approach has recently been expanded to localization of mRNA in the DRG cell body vs peripheral nerve fibers, in order to determine not only the expression, but also the sub-cellular localization of nociceptive TRP channel gene transcripts [83]. In contrast, information on expression and localization of these channels at the protein level is less conclusive. Antibody-based assessment of expression and localization of proteins has been the predominant approach, which is often inconclusive, due to inadequate/improper validation of specificity for these biological reagents. The nociceptive TRP channels are membrane proteins, and are localized to the neuronal/cell plasma membrane [84]. However, a significant fraction of channels, at least for TRPV1 and TRPA1 and TRPA1 have also been reported to be present in intracellular organelle membranes, which upon injury/inflammation undergo translocation to the plasma membrane [85,86,87,88]. Similar to the dearth of information on expression of other nociceptive TRP channels in the nervous system, their sub-cellular localization and modes of intracellular trafficking remain poorly understood. With the currently available information on nociceptive TRP channel expression and localization, it can be proposed that multiple pathological activators could activate TRPV1 and TRPA1 channels on the same sub-set of afferents, as well as on distinct TRPV1- or TRPA1-expressing sensory neurons. Overlapping expression of nociceptive TRP channels also provides an opportunity for cross-sensitization of TRPV1 and TRPA1 (example: by Ca^2+^) to maximize nociceptor excitation. In addition, inflammatory mediator-induced increases in the trafficking of TRPV1 and TRPA1 channels could increase the excitatory strength of nociceptors, referred to as “nociceptive tone”.

## 3. Modulation of Nociceptive TRP Channel Activity and Expression 

Nociceptive TRP channel activation on peripheral nerve fiber endings, at least for TRPV1 and TRPA1, has been directly linked to release of neuropeptides, such as calcitonin gene-related peptide (CGRP) and neurokinin or substance P (SP). Local elevation of CGRP and SP levels leads to vasodilatation and activation of a variety of immune cells, which results in the release of several pro-inflammatory mediators, growth factors, and bioactive peptides, as well as oxidative stress conditions. Most of these mediators activate specific G protein-coupled receptors (GPCRs) and growth factor receptors (GFRs) on sensory nerves, leading to downstream activation (or inhibition) of several protein kinases and phosphatases. These cellular signal transduction effectors induce post-translational modifications on multiple nociceptive channel proteins, leading to an increase in the activation of these channels (Figure 1), which results in an increase in nociceptor firing [9,11,13]. Specifically, modification of TRPV1 by protein kinases A and C (PKA & PKC), cyclin-dependent kinase-5 (Cdk5), *Src* kinase, and phosphoinositide kinases (PI3/4/5Ks) have all been shown to enhance activation of the channel by: (a) decreasing the temperature threshold of channel activation to physiological temperatures (~35 °C to 37 °C); (b) activating the channel at mildly/moderately acidic pH; and/or (c) enhancing plasma membrane delivery of the channel protein (Figure 1) [11,45,85,86,89,90,91,92,93,94,95,96,97]. Ca^2+^ influx through TRPA1 and subsequent activation of PKA has also been shown to modulate TRPV1 channel function [98]. In addition to kinases, lipases such as phospholipase C (PLC) have been shown to both promote and inhibit TRPV1 channel function. Hydrolysis of phosphatidylinositol bis-phosphate (PIP2) to inositol triphosphate (IP3) and diacylglycerol (DAG) by PLC sensitizes TRPV1 channel function via PKC, and by releasing constitutive inhibition of the channel from physical coupling with PIP2 [99]. In contrast, other studies have shown that plasma membrane PIP2 is necessary for TRPV1 channel activation [100]. Ca^2+^ influx through TRPV1 has been shown to activate protein phosphatase 2B (PP2B or calcineurin), which then dephosphorylates the channel protein to induce channel desensitization. Conversely, PKA phosphorylation of calcineurin-sensitive residues on TRPV1 protein has been shown to reverse this desensitization, thereby leading to sustained channel opening following activation [97]. All these changes culminate in constitutive activation of TRPV1 under pathophysiological conditions, thereby resulting in sustained nociceptor firing [9,11,13]. In contrast, GABA release from sensory afferents, downstream of TRPV1-mediated Ca^2+^ influx, has been shown to activate GABA_B_ receptors, leading to attenuation of NGF/serotonin/bradykinin-PKC-modulation of TRPV1 channel activity [101].

ROS/RNS and mechanical stimuli have been shown to activate TRPA1 [75]. Inflammatory mediators such as bradykinin have been shown to sensitize TRPA1 channel function [54], presumably via phosphorylation of the channel protein by PKC. However, another report suggested the involvement of PLC and PKA, but not PKC, in TRPA1 channel sensitization of TRPA1 [102]. Although the basis for such contrasting observations is not clear, it has been proposed that PLC/PKA-modulation of TRPA1 channel activation is achieved by increased trafficking of the channel protein to the cell plasma membrane (Figure 1) [87]. In addition to the modulatory actions on TRPV1 channel, PIP2 has also been shown to reduce the agonist sensitivity of TRPA1 channel activation [103]. Pro-inflammatory lipid metabolites such as lipoxygenases that are enriched during tissue injury and/or inflammatory conditions, have also been shown to activate TRPA1, in addition to TRPV1 [104]. Intracellular influx of Ca^2+^ itself has also been shown to be an intrinsic modulator of TRPA1 channel function [72].

Modulation of TRPM8 channel function has remained somewhat controversial, due to multiple conflicting observations. PKC has been shown to downregulate TRPM8 activity [105,106,107]; although one study did not find any effect of pharmacological inhibitors of PKC on TRPM8 channel function [108]. Unlike TRPV1 and TRPA1, TRPM8 function was found to be unaffected by PKA signaling [109,110,111,112]. Activation of Gα**_q/11_**-coupled receptors by various inflammatory mediators such as bradykinin, histamine, serotonin, and ATP, has been suggested to inhibit TRPM8 channel activity via direct interaction of the Gα**_q_** subunit with the channel protein (Figure 1) [113]. Such a phenomenon is thought to constitute a mechanism underlying reduced cold sensation under injury/inflammatory conditions. Modulation of TRPM8 channel function by endovanilloids and endocannabinoids have also been shown [114]. In addition, phospholipase A2 (PLA_2_) activity has been shown to enhance TRPM8 channel activity [115,116]. Furthermore, lipid metabolic products downstream of PLA_2_ activity, such as lysophospholipids have been shown to raise the temperature activation threshold of TRPM8 closer to body temperature [115]. An increase in PLC activity has also been linked to a decrease in TRPM8 channel activity [51,117,118,119].

Modulation of TRPV2 channel function by PKA has been shown in immune cells [120], although any role of such modulation in the context of nociceptor biology remains to be determined. Mediators of inflammatory signaling, lipid metabolites and PKC have also been shown to enhance TRPV3 channel activity [34,46], and similar to TRPV2, the role of such channel modulation in the context of nociceptor biology remains to be determined. Both PKA and *Src* phosphorylation of TRPV4, downstream of PGE_2_ and protease-activated receptor 2 (PAR2) signaling, have also been shown to modulate channel function [50,121,122,123]. In addition, PKC-dependent upregulation of TRPV4 channel activity has also been suggested [47]. All such modulatory actions of TRPV4 have been suggested to increase nociceptor firing in response to mechanical stimuli and/or osmotic changes. However, with a recent report suggesting no activation of TRPV4 in mouse DRG neurons to known chemical agonists and osmotic forces [80], the role of TRPV4 modulation in nociceptor function and AP firing remains to be confirmed.

In addition to direct functional modulation, upregulation of gene and protein expression of nociceptive TRP channels also serves as another mechanism for long-term modulation of nociceptor firing. Sustained intracellular Ca^2+^ influx/elevation due to prolonged/episodic activation of TRPV1 has been suggested to enhance the expression of several nociceptive TRP channel and related genes. TRPV1 expression (both at the mRNA and protein level) is enhanced in sensory neurons following tissue injury and inflammation [9,11,13]. Furthermore, an increase in the proportion of sensory neurons expressing functional TRPV1 channel has also been shown following injury/inflammation, as well as upon exposure to inflammatory mediators [9,11,13]. In addition, recent studies have shown rapid translation of TRPV1 mRNA in peripheral sensory fibers in response to pro-inflammatory mediators such as interleukin-6 (IL-6) and NGF, thereby increasing the magnitude of nociceptor excitation [124,125].

Altogether, modulation of nociceptive TRP channels by a plethora of inflammatory mediators provides diverse mechanisms for robust and sustained activation of these channels during multiple pathological conditions. An unified scheme could be proposed: Pathological conditions lead to: (1) local activation of TRPV1 and TRPA1 at physiological temperatures, mild-to-moderate acidic and oxidative stress conditions; (2) prolonged channel activation, due to reduced desensitization; (3) enhanced channel activation, due to increases in the expression and surface trafficking of TRPV1 and TRPA1 proteins; (4) cross-sensitization of channel activation (by Ca^2+^ and other intracellular signal transduction molecules), and (5) increased gene expression and local mRNA translation for these channels. Collectively, these processes result in an increase in nociceptive tone/excitation and prolonged nociceptor firing. These complex processes underlie mechanisms for peripheral sensory transduction, and provide both opportunities and challenges for the pharmacotherapeutic targeting of multiple painful pathologies.

## 4. Involvement of Nociceptive TRP Channels in Painful Pathologies

Nociceptive TRP channels have been shown to be involved in several pain-related pathological conditions/modalities, including inflammatory, neuropathic, visceral and dental pain, as well as pain associated with cancer [9,11,13]. Such information is mainly derived from numerous findings utilizing specific antagonists of individual nociceptive TRP channels in animal models of pain-related pathologies, as well as induction of such pathologies in mice with genetic deletion/alteration of individual nociceptive TRP channels. Both activation and/or modulation of nociceptive TRP channels during pathological conditions has been suggested to underlie the mechanisms associated with specific pain conditions, however, only a handful of direct in vivo evidence in support of these assertions is available.

### 4.1. Inflammatory Pain

In inflammatory pain conditions, the involvement of TRPV1 is the foremost and most well-established of all the TRP channels. Administration of small-molecule competitive antagonists of TRPV1 has been shown to attenuate thermal hyperalgesia induced by: (a) inflammatory conditions with administration of complete Freund’s adjuvant (CFA), formalin, zymosan etc.; and (b) local injection of individual inflammatory mediators in rodents [9,11,13]. Mice lacking the functional TRPV1 gene (*Trpv1^−/−^*) exhibit dramatic attenuation of thermal hyperalgesia in response to injection of a number of inflammatory mediators, with no alteration in noxious temperature responses observed in un-injected or saline-injected animals [9,11,13,126,127]. Similar results were also observed upon induction of cutaneous inflammation with administration of CFA, formalin, zymosan, etc. in *Trpv1^−/−^* mice. In addition to TRPV1, TRPM3 is also involved in the development of inflammatory thermal hyperalgesia, as *Trpm3^−/−^* mice demonstrate significant deficits in this pain modality [35]. The involvement of TRPV1 in mechanical hypersensitivity was initially ruled out, taking into consideration the initial findings from the phenotypic characterization of *Trpv1^−/−^* mice [126,127]. However, a large number of studies utilizing multiple animal models of inflammatory pain-like conditions have since suggested a critical role for TRPV1 in inflammatory mechanical hypersensitivity. These studies show attenuation of mechanical hypersensitivity by: (a) specific small molecule antagonists of TRPV1; and/or (b) administration of inflammatory mediators (or induction of inflammatory/disease conditions) in *Trpv1^−/−^* mice [85,128,129,130,131,132,133,134]. In addition to TRPV1, TRPA1 has also been proposed to be involved in inflammatory mechanical hyperalgesia. Pharmacological inhibitors of TRPA1 have been shown to attenuate CFA-induced mechanical hypersensitivity in rodents [135,136]. Furthermore, TRPA1 has also been suggested to mediate cold hyperalgesia under persistent inflammatory conditions in rodents [137,138]. Another aspect of the crucial role of TRPA1 in inflammatory pain states is the resultant inflammatory pain in chronic itch. It has been demonstrated that TRPA1 is critical to the development of neuropathic inflammation associated with allergic contact dermatitis [139]. TRPA1 has also been shown to be integral to the development of chloroquine-induced itch; in both transduction of itch and changes that occur in the skin associated with chronic itch [140,141]. In addition to TRPV1 and TRPA1 channels, the involvement of TRPV4 in inflammatory pain has also been proposed. In *Trpv4^−/−^* mice, increased latency to escape from hot plate following tissue injury and inflammation has been observed, which suggests a role for TRPV4 in the development of thermal hyperalgesia [142]. The role of TRPV4 in mechanical hyperalgesia, in responses to osmotic stimuli, both hypotonic and hypertonic, under injury/inflammatory conditions has also been proposed [49,50]. In osteoarthritis models, both TRPV1 and TRPA1 have been shown to play an important role. Genetic and pharmacological inhibition of TRPV1 can reduce arthritis-like symptoms [143,144,145]. In animal models both TRPV1 and TRPA1 activation results in increased release of TNF-α, a pro-inflammatory cytokine important for the development of osteoarthritis [145]. Further, there is evidence of TRPV1 involvement in osteoarthritis patient populations, including a TRPV1 variant associated with increased knee osteoarthritis, and increased expression of TRPV1 in the knee synovium of patients with osteoarthritis [144,146]. Additionally, topical capsaicin creams have long been used to relieve joint pain by desensitizing TRPV1-expressing nociceptors. Moreover, no edema or hypersensitivity were observed in *Trpv1*^−/−^ mice following joint inflammation, strengthening the assertion that TRPV1 is critically involved in the pathogenesis of arthritis-like inflammatory conditions [144].

Based on these pre-clinical findings, several small molecule antagonists of nociceptive TRP channels, mainly targeting TRPV1, have been tested in clinical trials for multiple inflammatory pain conditions, such as dental pain and osteoarthritis (Table 1). Most first-generation TRPV1 antagonists showed alteration in body temperatures, more specifically hyperthermia, and ultimately resulted in poor outcomes and/or termination of clinical trials [148,149,150,178]. In a phase II clinical trial, the TRPV1 antagonist AZD1386 failed to cause significant pain relief in patients with osteoarthritis [149,160], suggesting a more complex role for TRPV1, as well as involvement of other nociceptive channels/receptors in osteoarthritis. One positive scientific outcome from these failures in clinical trials can be attributed to the discovery and expansion of knowledge on the thermo-regulatory role of TRPV1. Furthermore, a clear and cautious warning has been relayed by these clinical findings—an in-depth basic scientific knowledge on these nociceptive ion channels/receptors is absolutely required before exploring these channels as clinical therapeutic targets. Therefore, a rational, specific, pathological function-based approach is required for targeting the modality-specific activation and/or modulatory properties of TRPV1 channel, in order to circumvent the thermoregulatory side effects. Recent development of drugs such as NEO06860 [162,163] and A1165442 [179], which are more potent antagonists of TRPV1 activation by capsaicin and protons, but not by heat, support these assertions. Contrarily, clinical development of a TRPV1 agonist, zucapsaicin, has been successful, and is currently in clinical use for osteoarthritis [171]. This drug is applied topically, which leads to excessive transient activation of TRPV1 on peripheral nerve fibers, followed by channel desensitization and/or nerve fiber degeneration. Whether the same outcome could be achieved by targeted attenuation of TRPV1 function on peripheral sensory nerves by a systemically administered small molecule antagonist in multiple inflammatory conditions remains to be elucidated. Other than TRPV1, modulators of other TRP channel have so far not been tested in clinical trials.

### 4.2. Neuropathic Pain

Neuropathic pain conditions result primarily from nerve injury due to structural damage and/or constriction, either related to neurological, viral and metabolic disease states or with the use of chemotherapeutic drugs. A number of different nociceptive TRP channels have been implicated in neuropathic pain states. Multiple studies utilizing TRPV1 antagonists in rodents and induction of neuropathy in *Trpv1*^−/−^ mice have demonstrated a significant role for TRPV1 in neuropathic pain, mainly associated with diabetes and chemotherapeutic drug use (reviewed in [9,47]).

Pharmacological inhibitors of TRPA1 have also been shown to be effective in attenuating mechanical hyperalgesia associated with neuropathic pain conditions [139,140]. TRPA1 can be activated by ROS/RNS, which is associated with several disease pathologies, one of which is diabetes [180]. Chemotherapeutic drugs such as oxaliplatin, vincristine and paclitaxel can also increase ROS/RNS production. Studies have shown that TRPA1 is critically involved in mechanical allodynia associated with such chemotherapeutic drug-induced neuropathic pain [181,182,183]. Cold hyperalgesia associated with nerve injury- and nerve ligation-induced neuropathic conditions in rodents have also been shown to require TRPA1, but not TRPM8, with pharmacological and antisense knock-down approaches [138,184]. Both TRPM8 and TRPA1 have been implicated in the cold allodynia associated with chemotherapeutic drug-induced neuropathic pain, although recent evidence suggests that it may primarily be confined to TRPA1 [185]. However, some studies have shown attenuated cold hypersensitivity responses in *Trpm*8^−/−^ mice in chronic constriction injury (CCI), and during the second phase of CFA injection [186]. Involvement of other nociceptive TRP channels in nerve injury/neuropathic conditions has not yet been established.

Based on various pre-clinical findings, small molecule blockers of TRPV1, TRPA1 and TRPV3 have been tested in clinical trials for various neuropathic pain conditions (Table 1). The only TRPV1 antagonist to enter phase II clinical trial*s* was AZD1386, which was subsequently terminated [161], and the underlying results/reasons were not published. More recently, phase II clinical trials on the TRPA1 antagonist GRC-17536 [174], and the TRPV3 antagonist SAR292833 [175] for neuropathic pain conditions have been completed. The results/outcome from these trials still remain unpublished. On the other hand, topical TRPV1 agonists, such as zucapsaicin and NGX 4010, have been quite successful in clinical trials for neuropathic pain conditions. NGX 4010 has already been launched for clinical use in human PHN-neuropathic pain conditions [164,165], and zucapsaicin has successfully completed phase II trials [173]. Therefore, it remains to be determined if antagonists of these specific nociceptive TRP channels could provide effective relief from neuropathic pain conditions. Furthermore, the site of action of drugs targeting nociceptive TRP channels, i.e., peripheral nerve fibers vs spinal cord, remains an area of concern, since central sensitization mechanisms operating at the level of spinal cord dorsal horn are highly critical in the maintenance of neuropathic pain conditions [9]*.*

### 4.3. Visceral Pain

TRPV1 is the most well studied nociceptive TRP channel in multiple visceral pain-like conditions. Studies utilizing pharmacological inhibition and genetic deletion of TRPV1 have been shown to decrease responses to colorectal distension in naïve and inflamed mice [187]. Further, studies have shown that inhibition of TRPV1 can decrease severity of disease in a variety of animal models for the initiation and maintenance of visceral hypersensitivity after injury [188]. TRPV1 has even been linked to severity of colorectal disease in human patients, where enhanced TRPV1 expression was positively correlated with disease severity [189]. To further link TRPV1 to visceral pain, multiple studies have shown that *Trpv1*^−/−^ mice show reduced pain-like behaviors in animal models of cystitis and inflammatory bowel disease [190,191,192]. While *Trpv1*^−/−^ mice demonstrate reduced pain-like behaviors during visceral inflammation, they show unexpectedly elevated levels of inflammatory markers compared to wild-type mice, which indicates that TRPV1 plays a more complex role in visceral sensitivity [193,194]. TRPV1 also plays a critical role in pain and alterations in bladder activity after inflammation [195,196,197]. Resiniferatoxin, a potent TRPV1 agonist that causes long lasting desensitization and/or degeneration of TRPV1-expressing nerve fibers, has been shown to decrease pain-like behaviors, as well as the number of bladder contractions in animals with bladder cystitis [196,198]. This evidence indicates that TRPV1 channel and TRPV1-expressing nerve fibers are vital to the development and maintenance of visceral pain. In addition to TRPV1, TRPV4 has been suggested to be involved in visceral pain hypersensitivity. Induction of pancreatitis and irritable bowel syndrome in *Trpv4^−/−^* mice lead to attenuated nociceptive responses when compared to wild-type mice, suggesting the involvement of TRPV4 in these visceral pain pathologies [199,200].

Several studies have also implicated TRPA1 and TRPM8 channels in visceral pain conditions. TRPA1 is expressed in a population of visceral nociceptors and it has been shown that pathological activation of TRPA1 induces neurogenic inflammation associated with irritable bowel syndrome and colitis, and the resultant inflammatory pain [201,202]. In combination with TRPV1, TRPA1 has been suggested to contribute to pain hypersensitivity downstream of PAR-2-stimulated pancreatitis [203]. The bacterial cell wall carbohydrate lipopolysaccharide (LPS) was found to activate TRPA1 directly, and was thus proposed as a mechanism for irritation/pain-like condition associated with bacterial infections [204]. In addition, TRPA1 has also been shown to be a critical component of chemosensory airway reflexes in response to irritants. TRPA1 on TG neurons has been shown to mediate sneezing and coughing reflexes [205]. Interestingly, *TRPA1^−/−^* mice do not exhibit the antinociceptive effects of acetaminophen and tetrahydrocannabinol (THC), suggesting a possible mechanism underlying analgesic properties of these drugs [206]. Peppermint oil, an agonist of TRPM8, can decrease pain in irritable bowel syndrome; however, these effects could be mediated by other channels, such as GABA_A_ receptors [207]. Additionally, there is some evidence that TRPM8 antagonists can reduce visceral pain-like behaviors in rats, such as overactive bladder and painful bladder syndrome [208]. So far, two TRPV1 antagonists have been tested in clinical trials for visceral pain conditions. The drug AZD1386 was tested in phase I clinical trials for esophageal pain conditions. This trial was completed; however the results/outcome still remain unreported [156,157]. The other TRPV1 antagonist SB-705498 entered into phase II clinical trial for rectal pain conditions; however, this trial was subsequently terminated and results have not yet been published. One of the major reasons for the slow progress of TRP channel antagonists in clinical trials for visceral pain conditions could be the lack of in-depth mechanistic studies on the involvement of nociceptive TRP channels in specific visceral pain conditions.

### 4.4. Pain Associated with Cancers and Other Pathological Conditions

#### 4.4.1. Cancer Pain

A number of studies have shown that TRPV1 plays an integral role in cancer pain. Specifically, studies have looked at cancer pain using rodent bone cancer models, and found that TRPV1 is critical for the development of cutaneous thermal and mechanical sensitivity, as well as paw guarding behavior [209,210]. There are many chemokines, cytokines and other factors that are released in the bone cancer microenvironment including prostaglandins, bradykinin, NGF, lysophosphatidic acid, and parathyroid hormone-related peptide, which have been shown to (or could presumably) sensitize TRPV1 currents leading to increased nociceptor firing [85,209,211,212,213]. Additionally, these mediators could potentially induce post-translational changes in TRPV1 and/or increase protein expression, which could lead to nociceptor sensitization. The tumor microenvironment is generally acidic, which could lead to sensitization of proton-activated TRPV1 currents [209]. Additionally, preclinical trials of intrathecal resiniferatoxin, the potent TRPV1 agonist, have shown it to be very effective in a canine model of bone cancer pain [168,214]. Based on these findings, phase I clinical trials have been initiated to determine the efficacy of periganglionic/intrathecal administration of resiniferatoxin in advanced cancer patients with bone pain [169,170]. Involvement of other nociceptive TRP channels in cancer pain has not been shown so far. One of the major challenges in this area has been the lack of pre-clinical models that more closely mimic the pathophysiology of advanced human cancer.

#### 4.4.2. Dental Pain

TRPV1 has also been linked to dental pain. Studies have shown that TRPV1 is expressed in 45%–85% of sensory neurons that innervate the tooth pulp, and increased expression of the channel has been reported in a rat model of pulpitis [215,216,217,218]. Although there is already strong evidence for the direct involvement of TRPV1 in dental pain, modulation of channel function by inflammatory/injury mediators could provide additional mechanistic understanding, which altogether makes TRPV1 an attractive target for pharmaceutical interventions. In support of enriched expression of TRPV1 on dental sensory nerves and its possible involvement in dental pain, multiple TRPV1 antagonists have been investigated in clinical trials. The TRPV1 antagonist AZD1386 was effective at providing relief for molar extraction pain compared to placebo, however the analgesia was not long lasting [159]. Other TRPV1 antagonists, GRC 6211, MK-2295 and SB-705498, have been investigated in phase II clinical trials [150,153,155], with two of these trials already completed [153,155]. However, the outcome/results from these studies still remain unpublished/un-reported. In addition to TRPV1, TRPA1 and TRPM8 have also been linked to dental pain, as they are expressed on many of the same fibers as TRPV1 in trigeminal neurons, however the precise mechanistic evidence in support of their direct involvement with dental pain is still lacking [217,219]. With the recent development and testing of TRPA1 and TRPM8 antagonists in pain conditions is likely to promote the testing of these drugs in clinical trials for dental pain conditions.

#### 4.4.3. Migraine

TRPV1, TRPM8 and TRPA1 have all been linked to migraine pathophysiology. Expression of TRPV1 and modulation of its function, trafficking and expression by multiple mediators in trigeminal neurons have been proposed to be involved in the development of migraine pathophysiology [10]. TRPV1 activation in neurons leads to release of CGRP, a critical neuropeptide in the development of trigemino-vascular excitation [10]. Accordingly, TRPV1 antagonists have been shown to be effective in alleviating migraine-like symptoms in rats [220]. Based on these basic and pre-clinical findings, a number of TRPV1 modulators have been investigated in clinical trials for migraine/headache conditions. So far, the only drug showing encouraging results is zucapsaicin, the channel agonist, for episodic cluster headache conditions [172]. Although a phase III clinical trial has been completed, the detailed outcome/results from this investigation still remain unpublished. Intriguingly, a gene variant of TRPM8 was discovered to have a positive correlation with migraine susceptibility in women [221]. However, it is not known whether this variant leads to any functional and nociceptive changes in TG neurons, and its direct involvement in migraine pathophysiology remains unexplored.

TRPA1 is expressed in trigeminal neurons and localized on dural afferents, where its activation has been shown to result in headache-like behaviors in mice [219,222]. Experimental drugs that serve as NO donors, such as nitroglycerine, have been extensively studied for their migraine-inducing properties. Both in humans and animals, NO directly activates TRPA1 via S-nitrosylation, and therefore, could serve as a mechanism for trigeminal excitation and migraine [8]. Interestingly, another recent study demonstrated the critical role of TRPA1 activation via monocyte/macrophage-induced oxidative stress conditions under trigeminal nerve constriction injury model in rodents [223]. Again, recent development of TRPA1 and TRPM8 antagonists for pain conditions is likely to promote testing of these drugs in clinical trials for specific migraine and headache conditions. Involvement of other nociceptive TRP channels in development of migraine and headache pathophysiology still remains unknown.

## 5. Nociceptive TRP Channels in Non-Painful Pathologies and Physiological Processes

Although characterized as critical detectors and transducers of nociceptive stimuli, these channels/receptors have also been implicated in several physiological and non-painful pathological conditions. TRPV1 has now been shown to play a role in body temperature regulation. Initial studies utilizing *Trpv1*^−/−^ mice suggested no involvement of TRPV1 in body temperature regulation [126,127]. During the process of drug development targeting TRP channels, it became clear that TRPV1 is critically involved in the regulation of body temperature, since systemic administration of small molecule antagonists of TRPV1 led to transient hyperthermia [147,148,149,150]. This in fact led to the failure of first-generation TRPV1-targeting drugs in clinical trials [147,148,149,150]. Subsequent studies have now suggested spinal cord TRPV1 as the critical mediator of noxious temperature detection [224]. In addition, TRPV1 in the brain stem has also been suggested to play an important role in thermoregulation, although expression and function of TRPV1 in the brain still remains a matter of debate [225]. TRPV1 has also been shown to exhibit high expression in sensory neurons innervating the airways, and is therefore involved in the cough reflex. Pharmacological blockade of TRPV1 has been shown to reduce cough in rodent models [226,227,228,229], supporting this assertion. Upregulation of TRPV1 protein expression has also been observed in asthma and gastro-esophageal reflux disease [226,228]. TRPV1 involvement in stomach cancers has also been suggested [228]. Interestingly, *Trpv1*^−/−^ mice exhibit increased sensitivity to insulin [230], raising the possibility of its involvement in diabetes. In fact, evidence suggests that TRPV1 activation could play a protective role in type I diabetes. However, other studies have suggested that activation of TRPV1 could be detrimental in type 2 diabetes conditions [228]. No significant contribution of TRPV2 has been suggested in pathological pain and nociceptive signal processing in vivo, as substantiated by observations utilizing *Trpv2*^−/−^ mice, which display normal thermal and mechanical nociceptive behaviors [231]. However, TRPV2 has been proposed to influence macrophage function and phagocytosis [232].

In addition to TRPV1, involvement of TRPM8 in thermoregulation has also been suggested, although in the opposite direction. Deficiencies in cold sensation were observed in *Trpm*8^−/−^ mice, with a decrease in avoidance behavior to moderately cold temperatures was observed in these animals [186,233,234]. Additionally, a selective TRPM8 antagonist showed no significant alteration in body temperature when administered to healthy volunteers, further suggesting it may not be a critical regulator for the maintenance of body temperature [176]. Avoidance of cold temperatures (below 0 °C) could still be observed in *Trpm*8^−/−^ mice. This could be due to other cold sensitive channels, or any compensatory changes due to specific deletion of the *Trpm8* gene. In this regard, a role of two other channels has been proposed: (a) leak K^+^ channels, such as TRAAK and TREK1, which close at very low temperatures; and (b) TRPA1, which is sensitive to noxious cold temperatures [31,235]. In spite of the important role of TRPM8 in cold hypersensitivity, this channel has also been suggested as the mediator of cold- and menthol-induced analgesia. Cooling, as well as administration of menthol has been shown to reduce acute and inflammatory pain in rodent formalin injection pain models. Interestingly, such analgesic effects of mild cooling are absent for the inflammatory pain phase after formalin injection, but still present for the acute phase in *Trpm*8^−/−^ mice [233]. Apart from the well-defined role of TRPA1 in pain and cold hypersensitivities, this channel has also been suggested to play a critical role in vertebrate hair cell mechanotransduction and hearing [236,237]. However, subsequent studies utilizing targeted deletion of the *Trpa1* gene in mice showed no involvement of TRPA1 in hair cell mechanotransduction [238]. TRPA1 has been shown to be a sensor of wide range of environmental irritants and proalgesic agents (reviewed in [239,240]).

## 6. Concluding Remarks

Identification and cloning of nociceptive receptors, mainly belonging to the TRP channel family, in the last decade and-a-half has tremendously advanced our understanding of the biology of nociception and multi-modal pain sensation. In-depth characterization of functional properties of these receptor channels, and describing their expression in various tissue and individual cell types within the nervous system has pushed us closer to connecting all the dots of somatosensory, visceral and trigeminal sensory pathways. In addition, extensive utilization of mouse genetics in sensory biology has been incredibly helpful in this process. Simultaneous development of pharmacological interventions targeting nociceptive TRP channels has not only been closing in on new-generation analgesic drug developments, but also providing vital information on the in vivo mechanisms of sensory signal processing. Although there is still a great deal to uncover in the biology of nociception, the startling and relatively recent progress in expansion in knowledge in this area of research will undoubtedly lead to more efficacious and evidence-based management of multiple pain pathologies.

## Figures and Tables

**Table 1:** Small molecule modulators of TRP channels as drugs in clinical development.

Agonist/Antagonist (Producer/Company)	Channel (Mode of Action)	Current Clinical Use/Trial Status	Specific Painful Pathology	References
AMG517 (Amgen)	TRPV1 (channel blocker)	Phase Ib/Phase II-terminated	Dental pain	[147,148,149,150]
ABT102 (Abbott)	TRPV1 (channel blocker)	Phase I—completed; Phase II—unknown	Healthy volunteers	[148,149,150,151,152]
GRC 6211 (Lilly/Glenmark)	TRPV1 (channel blocker)	Phase I-Phase II	Dental pain	[148,149,150]
SB-705498 (GlaxoSmithKline)	TRPV1 (channel blocker)	Phase II—completed	Dental pain/Toothache	[149,150,153]
		Phase II—terminated	Rectal pain	[154]
MK-2295 (Merck-Neurogen)	TRPV1 (channel blocker)	Phase II—completed	Postoperative dental pain	[149,150,155]
AZD1386 (Astra-Zeneca)	TRPV1 (channel blocker)	Phase I—completed	Esophageal pain	[149,150,156,157]
		Phase II—completed	Dental pain	[149,158,159]
		Phase II—terminated	Osteoarthritis, knee pain	[149,150,160]
		Phase II—terminated	Neuropathic pain	[161]
NEO06860 (Neomed Institute)	TRPV1 (blockade of channel activation by capsaicin, but not by heat and protons)	Phase I—completed Phase II—ongoing	Osteoarthritis, knee pain	[162,163]
NGX 4010 (NeurogesX; Acorda Therapeutics/ Astellas Pharma)	TRPV1 (agonist; capsaicin transdermal patch)	Phase III—completed (launched for clinical use in PHN)	PHN-associated neuropathic pain	[164,165]
		Phase III—completed	HIV-associated neuropathic pain	[165,166,167]
Resiniferatoxin (NIDCR-NIH)	TRPV1 (potent agonist)	Phase I—ongoing	Advanced cancer pain	[168,169,170]
Zucapsaicin (Sanofi-Aventis; Winston Pharmaceuticals)	TRPV1 (agonist; nasal and topical capsaicin patch & cream)	Phase III—completed (launched for clinical use in osteoarthritis)	Osteoarthritis, knee pain	[171]
		Phase III—completed	Episodic cluster headache	[172]
		Phase II—completed	PHN-associated neuropathic pain	[173]
GRC-17536 (Glenmark)	TRPA1 (channel locker)	Phase II—completed	Diabetic peripheral neuropathic pain	[174]
SAR292833 (Sanofi)	TRPV3 (channel blocker)	Phase II—completed	Neuropathic pain	[175]
PF-05PR105679 (Pfizer)	TRPM8 (channel blocker)	Phase I—completed	Healthy volunteers	[176,177]

For an extended list of drugs targeting TRP channels and their development status, refer to earlier reports [150,178].
